# Measuring Wellness Through Indigenous Partnerships: A Scoping Review

**DOI:** 10.3390/ijerph22010043

**Published:** 2024-12-31

**Authors:** Lynn Mad Plume, Danya Carroll, Melanie Nadeau, Nicole Redvers

**Affiliations:** 1 Department of Indigenous Health, University of North Dakota School of Medicine and Health Sciences, Grand Forks, ND 58202, USA; lynn.madplume@und.edu (L.M.P.); melanie.nadeau@und.edu (M.N.); 2 Schulich School of Medicine and Dentistry, University of Western Ontario, London, ON N6G 2M1, Canada; dcarro4@uwo.ca

**Keywords:** Indigenous Peoples, measures of wellness, scoping review, Indigenous measures of wellness, Indigenous partnerships, community engagement, co-development research, Indigenous methodologies, Indigenous wellness, research partnerships

## Abstract

Indigenous wellness has been defined in varying contexts by diverse Indigenous Peoples. The existing indicators used to measure wellness are often defined from a Western perspective. Despite the rich conceptualizations of Indigenous wellness, there exists a notable gap in how it can be measured in contemporary contexts through an Indigenous lens. A scoping review methodology with the aim of identifying measures of wellness developed through Indigenous partnerships was carried out. We completed a systematic search in the following electronic databases: PubMed, CINAHL, Psych Info Academic Search Complete, SocIndex, and the Native Health Database. We then carried out a two-stage article screening process to identify eighteen relevant papers. Content analysis was then used to identify (1) the major categories for the partnership contexts utilized in the process for measuring Indigenous wellness and (2) the kinds of measures developed. Five main categories were characterized, including the following: (1) building relationships that uphold Indigenous worldviews is important, (2) a call for co-development protocols that weave multiple worldviews, (3) the need to increase awareness of the limitations in measuring Indigenous wellness, (4) community-specific context is important, and (5) a call for strengths-based indicators. Governments, organizations, and research partners are called upon to support the co-development of meaningful engagement protocols that privilege and reflect Indigenous voices and perspectives when measuring Indigenous wellness.

## 1. Introduction

Wellness indicators have been used for decades by various governments and organizations to measure the health of Indigenous Peoples around the globe [1]. Health assessments conducted by government agencies and researchers from outside Indigenous communities often lack basic methodological guidance on how to respectfully and appropriately evaluate Indigenous wellness, which can compromise the quality and consistency of results [1,2]. The concept of wellness used to inform health data collection is not well defined, and most often reflects non-Indigenous standards for quantifying Indigenous wellness [3]. More specifically, misaligned indicators and measures of wellness have been widely applied in Indigenous contexts, which has been to the detriment of the health of Indigenous Peoples [4,5]. For example, Indigenous Peoples often consider the wellness of the whole community and their surrounding environments, such as their connection to Land and engagement with family, community, and traditional activities as part of their conceptualizations of wellness [1,5]. In contrast, the Western view and model of health most often adopts a biomedical perspective that isolates wellness from other interrelated elements (e.g., the health of the environment) [5,6]. Despite the efforts of some researchers in various health contexts to address this discrepancy in how wellness is viewed and measured, Indigenous health inequities continue to persist and be contextualized through a Western lens [4,7].

Historically, Indigenous research has been completed on, rather than with or by, Indigenous Peoples [8], and often uses research methods that are not inclusive of Indigenous knowledge or ways of knowing [8,9]. Although there has been some positive progress over the years, current research practices still tend to encapsulate extractive practices that uphold exploitative partnership processes [10,11]. Indigenous Peoples continue to be the subjects of research endeavors, rather than consenting participants [9], and are much less likely to be considered as research partners. Indigenous Peoples have reclaimed space within research realms in recent years, reimagining their relationship with research and researchers through greater self-determination and involvement in research governance [11,12]. For example, Indigenous Data Sovereignty refers to “the right of Indigenous Peoples to govern how data from or about them is collected, accessed, used, stored, and disposed of—thereby repositioning Indigenous authority over Indigenous data away from an extractive, colonial model” [12]. Indigenous Data Sovereignty has sparked talks on restructuring research data practices away from colonial frameworks and platforming the significance of Indigenous Data Governance within Indigenous communities for making decisions and managing research data [9,12].

Despite this increasing shift in research practice to one that is with and by Indigenous Peoples, Indigenous wellness research continues to be fraught with misaligned measures and processes for developing measures [10,13]. For Indigenous Peoples, the idea of wellness is closely linked to their identities, cultures, and ways of life, significantly influencing both their physical and spiritual wellbeing [1,13]. Given this more holistic view of wellness, in addition to the diversity of Indigenous Peoples spanning the globe, there is a need to have a deep respect for the cultural nuances within Indigenous communities that might impact the wellness measures used [3,14].

For a better appreciation of the cultural nuances within a respective Indigenous community, meaningful partnerships and collaboration throughout the research process are essential [9,11,13]. This type of meaningful partnership can and should be inclusive of the initial stages of identifying relevant wellness measures, the data collection phases, analysis, the interpretation of the results, and the sharing of the results with those who the research reflects [10,15]. Developing meaningful and ongoing partnerships with Indigenous Peoples has the potential to transform wellness research through shared learning and the co-development of meaningful engagement protocols [15,16]. Genuine community engagement entails engaging in collaborative efforts with those who share similar experiences, concerns, or challenges; fostering partnerships to mobilize resources and influence systems; and catalyzing changes in policies and practices [17].

Working towards more equitable, sustainable, and healthy futures for Indigenous communities requires measures of wellness to be informed and guided by different knowledge paradigms, including Indigenous Knowledge [16,18]. With this, the overall goal of our scoping review was to systematically gather and analyze the published literature to identify common approaches for engaging Indigenous populations in conversations on measures of wellness. More specifically, our review aimed (1) to identify from the literature measures of wellness that have been developed through Indigenous partnerships, (2) to identify the processes of Indigenous community engagement used when developing or translating measures of wellness, and (3) to reflect on identifiable research gaps.

### Positionality

There has been a significant call for increased acknowledgment and recognition of Indigenous knowledge systems and methodologies in academic settings [12]. Indigenous research methodologies emphasize the critical importance of the positionality of individuals who write about, with, for, or by Indigenous Peoples [8,18,19]. Therefore, the authors of this review position themselves as Indigenous health scholars committed to working to improve the health outcomes of Indigenous Peoples and communities. The first author (L.M.P.) is a member of the Amskapi Piikani (Blackfeet Nation) in the United States (US). The second author (D.C.) is from the Diné and White Mountain Apache Tribal Nations in the US, and the third author (M.N.) is a member of the Turtle Mountain Band of Chippewa Indians, also in the US. Finally, the senior author (N.R.) is a member of the Deninu K’ue First Nation from Denendeh in northern Canada. This positioning allows us to approach research with a deep understanding of Indigenous perspectives and values, fostering meaningful collaboration and promoting culturally responsive methodologies. Through this lens, we aim to contribute to the advancement of Indigenous wellness and health outcomes.

## 2. Materials and Methods

This scoping review was conducted in accordance with the Arksey and O’Malley Methodological Framework [20], further refined by Peters et al.’s [21] methodology for scoping reviews. The Preferred Reporting Items for Systematic Reviews and Meta-Analyses extension for Scoping Reviews (PRISMA-ScR) checklist was used to guide the reporting [22]. A scoping review protocol was registered on the Open Science Framework (OSF) on 08 June 2023 [23].

### 2.1. Review Question

The scoping review procedure, including the search strategy and the screening and selection criteria, was guided by the following review question: What is known from the literature about the measures of wellness that have been developed or translated through Indigenous partnerships?

### 2.2. Search Strategy

The development of the search strategy was conducted in consultation with a research librarian at the University of North Dakota School of Medicine & Health Sciences (see File S1 for example search terms). The following electronic databases were searched for relevant articles: PubMed, CINAHL, PsychInfo Academic Search Complete, SocIndex, and the Native Health Database. In addition, manual searches were conducted in the *International Journal of Indigenous Health*, *Journal of Indigenous Wellbeing*, *International Indigenous Policy Journal*, *American Indian Culture and Research Journal*, *Turtle Island Journal of Indigenous Health*, and the Indigenous Studies Portal (iportal). Additionally, a search was conducted via Google and Google Scholar using adapted search terms based on the inclusion criteria until there were two pages with nothing of relevance found. Lastly, a manual search of the reference lists of key articles was conducted to further identify any additional articles that met the inclusion criteria.

### 2.3. Screening and Selection

All articles identified in the search strategy were exported into the Covidence (v2721 a9510157) [24] review software to facilitate the article screening process. Due to a potential lack of research on engagement and partnership approaches that are relevant to Indigenous Peoples for measures of wellness, there was no limit set for the article publication dates to ensure the broadest scope of included literature. Articles were searched from English-language publications, including project or research work, theses or scholarly pieces, government and organizational publications, and open-access book chapters. Non-English articles, text or opinion papers, non-open-access book chapters, and print books were excluded due to constraints related to translation, budget, and time.

Articles were included if the projects or initiatives aimed to engage and partner with Indigenous Peoples to develop or assess Indigenous measures of wellness. Measures were seen to be narrow in focus with any number of quantitative measures, general indicators, or instruments seen to be relevant for this review. Although there are many ways to consider the measurement of wellness within Indigenous communities that are broader than our review criteria, we felt that it was important to hone in on a specific sub-set of measures that are not often considered together. The initial search was conducted up to 19 June 2023, with an updated search being carried out up to 11 October 2024. Based on the United Nations’ use, this review defines Indigenous Peoples as “communities that live within, or are attached to, geographically distinct traditional habitats or ancestral territories, and who identify themselves as being part of a distinct cultural group, descended from groups present in the area before modern states were created and current borders defined” [25]. For the purpose of this review, the concept of Indigenous wellness was seen to be in alignment with the First Nation Health Authority (FNHA) Framework of First Nation Perspectives of Health and Wellness [26]. Table 1 provides an outline of Indigenous wellness concepts adapted from the First Nation Health Authority’s First Nation Perspectives of Health and Wellness [26]. With this, for the purposes of this review, wellness is defined as “a whole and healthy person [or peoples] expressed through a sense of balance of spirit, emotion, mind and body. Central to wellness is [the] belief in one’s connection to language, land, beings of creation, and ancestry, supported by a caring family and environment” [14,16,27]. Platforming this definition of wellness ensured that the selected articles aligned with Indigenous views on wellness.

Given the focus of this article, it is critical to explicitly define how we examined community engagement in our review. We defined community engagement as engagement that involves the development of partnerships that mobilize resources and influence systems, that build relationships among partners, and that serve as catalysts for changing policies, programs, and practices [16]. Setting the community engagement context was necessary to ensure that the perspectives and needs of Indigenous communities were flagged when reviewing articles for the Indigenous partnership criteria. With this, we only included articles that demonstrated an Indigenous community engagement context throughout the project.

A two-phased screening process was conducted to determine article inclusion. During the first phase, the titles and abstracts of all articles identified through the search strategy were screened by two authors independently (L.M.P. and D.C.), and any discrepancies were resolved by discussion with a third reviewer (N.R.). During the second phase, one author (L.M.P.) screened all of the full-text articles identified in the first phase, with a second author (D.C.) screening 10% of the articles to ensure consistency. A third author (N.R.) was brought in to resolve any discrepancies by discussion.

### 2.4. Data Extraction and Analysis

One author (L.M.P.) carried out data extraction from the included full-text articles, with a second author (D.C.) cross checking a random sample of 10% of the articles. The data were then charted in Microsoft Excel 365, and included the following information: article title, general article information (citation, year, country, and funding [if specified]), and study details and characteristics (objective, design, data collection method, setting, population, and Tribe [if specified]) (see File S2). The included articles were analyzed using directed qualitative content analysis (DQICA) [28] with NVivo (Release 14.23.2), a qualitative data analysis software tool, to identify measures of Indigenous wellness and the partnerships involved in developing these Indigenous measures of wellness. For the directed qualitative content analysis, our review followed the process outlined by Kibiswa [28], which has three main phases, including preparation, organization, and reporting [28]. During the first phase, the authors developed a guiding framework and operational definitions based on previous measures of wellness that were developed through Indigenous partnership projects. The included articles were then reviewed to identify high-level categories. During the second phase, one author (L.M.P.) coded and organized the data using NVivo (Release 14.23.2), a qualitative software based on the previously identified categories. During the third phase, one author (L.M.P.) used the NVivo qualitative software to organize and identify the key categories identified through phases 1 and 2, which guided the reporting of the results. Since this review aimed to gain a broad perspective of the topic area, inclusive of both empirical and grey literature, and was not based on assessing for specific outcomes, a quality assessment of the included articles was not conducted.

## 3. Results

**Figure 1:**
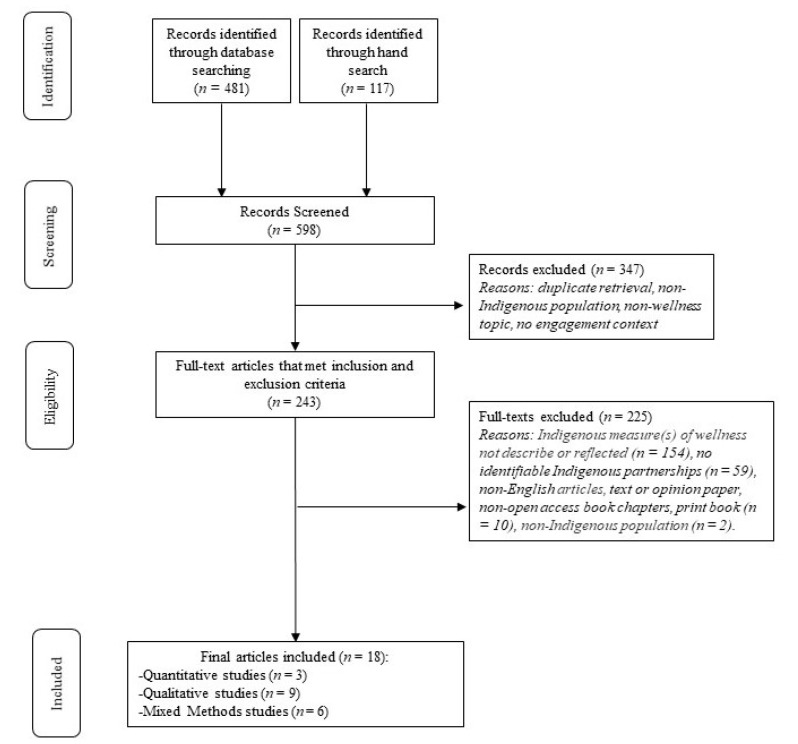
Adapted PRISMA diagram.

Through the systematic search, eighteen relevant articles were identified that met the inclusion criteria (see Figure 1). The majority of projects noted within the included articles recruited participants from an Indigenous Nation or community (*n* = 17), and only one included a more global meta-level perspective of wellbeing measurement efforts. A few of the projects developed a framework to inform the measurement of Indigenous wellness (*n* = 6) [29,30,31,32,33,34], and a few projects identified indicators or measures of wellness (*n* = 4) [35,36,37,38]. Some of the projects developed and validated a measurement tool (*n* = 3) [38,39,40], whereas other projects validated an existing tool (*n* = 2) [41,42] or developed a new tool (*n* = 3) [43,44,45]. Over half of the included articles had projects that were carried out in Australia (*n* = 10), with the remaining projects being conducted in Canada (*n* = 4), New Zealand (*n* = 2), and the United States (*n* = 2).

The majority of the included articles (*n* = 17) were published after 2013, with the earliest article being published in 2006 [35]. Also noted was an authorship overlap in four of the included studies [30,34,38,43]. The majority of the studies utilized qualitative designs (*n* = 9), followed by mixed-methods design (*n* = 6), with the remaining articles utilizing a quantitative method design (*n* = 3). Focus groups were the most used form of data collection method (*n* = 12) across the studies, with an Indigenous method (i.e., “yarning”) used within five of the articles published from projects in Australia [37,39,43,45,46]. Key informant interviews and surveys additionally informed ten of the included articles, with only one article utilizing a photovoice approach for data collection [44]. A couple of the included articles (*n* = 2) reported young people as participants in the project [41,44]. For an outline of the data characterization of the included studies, see Table 2, with a more detailed extraction table available in File S2.

### 3.1. Measuring Indigenous Wellness Through Meaningful Partnership

Indigenous perspectives and sovereignty were central to the characterization of Indigenous wellness indicators, with calls for approaches grounded in Indigenous worldviews and values. Participatory research methodologies were often used in supporting communities in these Indigenous approaches while fostering ownership and agency in the measurement of wellness [30,36,37,38,39,40,43]. Key findings advocated for genuine community control over Indigenous wellness indicator development, ensuring that measures resonated with cultural values and lived experiences [33]. There were also a number of key findings which emphasized the importance of community engagement in developing partnerships, as well as the need for measures of wellness to be culturally relevant [29,45,46]. There was emphasis placed on the importance of strengths-based approaches, meaningful partnerships, and the creation of wellbeing frameworks that embody more-than-human wellbeing, are inclusive of diverse worldviews, and are co-developed with Indigenous Peoples [30,31,33,34]. The importance of these approaches and partnership styles was in ensuring holistic and culturally responsive approaches to wellness measurement and service delivery.

Through directed qualitative content analysis [28], we identified five overarching categories for engaging Indigenous populations in conversations on measures of wellness through Indigenous partnerships (see Table 3). These five overarching categories included the following: (1) building relationships that uphold Indigenous worldviews necessitates community-driven processes for measuring wellness, (2) there is a need to move towards co-development engagement protocols that weave multiple worldviews, (3) awareness is needed about some of the limitations in measuring wellness within Indigenous community settings, (4) community-specific context matters when developing Indigenous indicators and measures of wellness, and (5) there is a need to shift to strengths-based Indigenous wellness measures and indicators. These categories are further described below in further detail.

#### 3.1.1. Building Relationships That Uphold Indigenous Worldviews Necessitates Community-Driven Processes for Measuring Wellness

Some of the included articles noted that Indigenous communities often report that a shared perspective of wellness is critical and requires a two-way or shared learning process [30,33,34,45]. For example, research by Cairney et al. [30] highlighted that “Community members expressed their lack of knowledge and confidence about how these ‘whitefella ways’ [i.e., Western knowledge and governance systems] work and their eagerness to learn more from non-Aboriginal people in two-way models of learning [30]. Other included studies also noted that recognizing the importance of community context and collaborative indicator development acknowledges the diversity of health indicators across communities while emphasizing the need for a core set of comparable indicators in measuring Indigenous wellness [30,34,41,43]. The process of defining these indicators should involve diverse community rights-holders to honor local perspectives and ensure relevance [31,33,38,43].

A number of authors recognized that wellness transcends the personal sphere, encompassing an understanding of interconnectedness where healing and wellness thrive through relationships [31,33,35,42,46]. It was noted that Indigenous Peoples seek harmony not only within themselves, but also in the relationships between people, culture, and spirit [31,33,36,42,45]. Indigenous wellness research conducted within a community-specific context should include Indigenous conceptualizations of wellness and emphasize the importance of balance among various realms of wellness (e.g., physical, spiritual, and emotional) as a central indicator [31,33,41,42]. Research partners can uphold Indigenous worldviews by necessitating community-driven processes for measuring wellness, ensuring cultural authenticity, relevance, and local ownership while acknowledging power dynamics [29,31,35,37,41]. Several of the included articles also highlighted that, for such community-driven processes to be carried out, employing Indigenous research methodologies was optimal [34,38]. Utilizing a culturally rooted approach aligned with Indigenous traditions, fostering conversations that supported the exchange of stories and knowledge [33,35,39,40].

A few of the included studies emphasized that meaningful collaboration within a community context should not be seen as a means to an end, but rather as a pathway to achieving enduring, sustainable benefits by fostering deeper relationships and community inclusion [29,31,34,43]. Some authors also highlighted that relationships, in general, hold paramount importance within research work around measures of wellness, with all forms and levels of relationships being crucial to considering wellness [31,34,43]. A focus on relationships and their associated impacts on wellness, rather than on indicators around individual wellness separated from the community and relational context, was stated to be important [29,31,33,36].

#### 3.1.2. There Is a Need to Move Towards Co-Development Engagement Protocols That Weave Multiple Worldviews

The included articles highlighted the importance of moving towards co-development engagement protocols that weave multiple worldviews [29,30,33,37,43]. Approaches that centralized Indigenous Peoples and their worldviews as integral to solutions, rather than passive recipients of Western ideology and health practice, were noted to contribute to movements of decolonization and the reclamation of Indigenous identity and knowledge as assets [35,37,39,40,41]. Co-developed engagement protocols within Indigenous wellness research contexts were noted to resonate deeply with Indigenous Peoples, as they maintained control over their narratives and the manner in which their narratives were shared throughout the research process [30,33,43]. Colonization was stated to often lead Indigenous Peoples to report unequal partnerships within research contexts and settings [29,30,32,37,42]. For instance, in a study in Australia investigating culturally relevant wellbeing indicators among Yolngu Indigenous Peoples, concerns were voiced regarding imbalances in the levels of control and respect between Balanda (non-Aboriginal people) and Yolngu (Aboriginal people) [30]. Yolngu Peoples emphasized the importance of achieving parity in control, respect, and reciprocal knowledge exchange, advocating for collaborative and equitable relationships [30]. They expressed dissatisfaction with the prevailing imbalance where Balanda (non-Aboriginal people) often hold greater control, underscoring the necessity for mutual respect and shared learning [30]. Yolngu Peoples highlighted the desire for joint training sessions and collaborative work on an equal footing, stressing the importance of working together and supporting each other [30].

Additional included articles underscored that shared learning fosters equal partnerships in Indigenous wellness research, exemplifying meaningful collaboration and inclusivity in co-development processes [29,30,33,37,43]. In this review, the included articles highlighted the transformative potential of co-development processes in bridging cultural divides and leveraging collective wisdom to effectively address complex challenges [29,37,39,42]. Several of the included articles suggested that co-development initiatives provided a unique opportunity to strengthen the connections between Indigenous cultural leadership and mainstream leadership, and also promoted equal partnerships over emphasizing differences [31,35,39,45]. Through co-development engagement protocols that weave multiple worldviews, research partners were stated to potentially narrow the gap between different worldviews, fostering mutual respect and understanding [29,30,33,37,43,46]. Many of the included articles stated that co-development processes that weave multiple worldviews, such as yarning, were culturally grounded and conducive to Indigenous ways of doing [37,43,45,46]. Furthermore, co-development strategies that engage non-Indigenous peoples and Indigenous Peoples in conversation to share stories and exchange knowledge were viewed as optimal for weaving worldviews [30,41,43].

#### 3.1.3. Awareness Is Needed About Some of the Limitations in Measuring Wellness Within Indigenous Community Settings

In this review, the included articles emphasized the significant implications of excluding and misunderstanding Indigenous perspectives on wellness within policy frameworks. The omission of Indigenous perspectives limited health and wellness policy effectiveness [31,34,36,42,43] and was noted to perpetuate unequal power dynamics between Indigenous and non-Indigenous peoples [31,34,39]. Conventional wellness indicators misidentified Indigenous identity as a health risk, neglecting the deeper impacts of colonization, historical trauma, and the social determinants of health [29,34,36,41,43]. The included articles noted that excluding and/or misunderstanding Indigenous wellbeing narrowed various governing entities’ ability to develop appropriate policies, which thereby limited the policy’s effectiveness [31,34,38]. Decisions made outside the context of Indigenous communities were found to have far-reaching implications for internal community dynamics, highlighting an unequal power balance between Indigenous and non-Indigenous populations [29,34,36,41,43].

Projects conducted within a community partnership context identified that misaligned Indigenous wellness indicators often perceived Indigenous identity as a risk factor for poor health [34], rather than recognizing colonization, ongoing and historical trauma, and the social determinants of health as contributing factors to health disparities within Indigenous communities [29,34,36,43]. One article stressed that misaligned Indigenous wellness measures made it difficult for governments to justify resource allocation [31]. Some authors argued that Western models of wellness measurement focus solely on disease symptoms, neglecting the broader social, economic, political, or spiritual contexts within communities [29,32,35]. An article by Peters et al. also noted that excluding and misunderstanding Indigenous perspectives of wellness leads to “prescriptive stereotypes, an external locus of control, learned helplessness, and a self-fulfilling prophecy, which perpetuates these health disparities” [41]. Indigenous wellness research conducted within other community partnership context suggested that indicators of Indigenous wellness must advance to encompass a holistic view grounded in interconnected relationships and community-specific context [33,34].

The meaningful operationalization of Indigenous wellness indicators was found to require community involvement in crafting, formulating, and investigating these indicators to align with community objectives and aspirations for a healthy community [30,33,36]. A few articles noted that employing a solely ‘one-size-fits-all’ indicator approach is not feasible at the community level [35,39]. This one-size-fits-all approach was not feasible due to the contextual nuances of relationships that exhibit distinctive traits specific to each local community [35,39]. Overall, many articles concluded that effectively applying Indigenous wellness indicators requires clarity on several key aspects, as follows: the reasons for measuring wellness, how wellness is conceptualized, the processes used to define wellness measures, and who has control over the decisions regarding these measures [31,35,37]. Lastly, the limitations to understanding Indigenous wellness were noted to be addressable at the community level through the co-development of engagement strategies that promote collaborative leadership, trusting relationships, and shared power [31,34,43].

#### 3.1.4. Community-Specific Context Matters When Developing Indigenous Indicators and Measures of Wellness

Indigenous wellness research conducted within a community-specific context was noted within the included articles to be significant in the development of Indigenous indicators and measures of wellness [35,38,39,40]. The approach of developing indicators at the community level was emphasized to require a profound respect for the community and its members [29]. Projects specifically underscored the importance of respecting Indigenous ways of knowing and the community-specific context [29,34,43]. Articles included within the review emphasized that understanding the community perspective and reporting the community’s understandings of wellness in their own words were essential [29,36,39]. The centrality of kinship in defining and conceptualizing indicators of Indigenous wellness was also highlighted. In particular, the involvement of Elders in decision-making processes was noted, which was seen to foster vital relationship building [43]. Some articles highlighted that including Elders in decision-making processes accentuated the importance of prioritizing relationships while fostering reciprocal bonds between Indigenous and non-Indigenous partners [36,39,40,42].

Creating culturally relevant population wellness measures was seen to be as equally important as involving community members in identifying specific indicators that reduced the risk of disempowerment [35,38,39]. Projects stressed that there is much diversity among Indigenous Peoples, which necessitates coming to a shared understanding of wellbeing that is meaningful to a respective Indigenous community [29,30,34,43]. Having a shared understanding of wellbeing better ensures validity and reliability through culturally specific interpretations of wellness at the community level [29,30,36,43]. The included articles also emphasized the importance of employing Indigenous research methodologies for community-driven processes, as this allowed for the aligning with Indigenous traditions and fostered conversations resulting in the exchange of stories and knowledge [31,37,39,40,41,43,46].

A few articles noted that Indigenous Peoples often perceived significant power imbalances between local Indigenous communities and non-Indigenous community members [29,30,33,37,43]. Non-Indigenous community members were observed to have a greater influence inn decision-making processes and superior access to resources and services, which could impact the way that measures are developed [30,31]. Improving Indigenous Peoples’ health necessitated recognizing the significant power imbalances that persist between local Indigenous communities and non-Indigenous community members. Continuing to neglect the co-development of engagement protocols and not fostering shared understandings could impede progress towards the greater wellbeing of Indigenous Peoples [29,30,33,37,43]. With this, acknowledging power dynamics, ensuring the co-development of engagement protocols, and fostering shared understanding in research processes are critical for developing Indigenous wellness measures [30,31]. Developing objective indicators that authentically represent the values and priorities of Indigenous communities was stated to be crucial for localized acknowledgment and advocacy by communities, researchers, governments, and industries [35,38].

#### 3.1.5. There Is a Need to Shift to Strengths-Based Indigenous Wellness Measures and Indicators

Many of the included articles stated that building relationships that uphold Indigenous worldviews requires a shift to strengths-based Indigenous wellness measures and indicators [29,30,32,37,42]. Some authors noted that non-Indigenous and deficit-based approaches to wellness measurement uphold an incorrect assumption that the adoption of mainstream values and practices leads to an improved quality of life for Indigenous Peoples [29,32,33,44]. The lack of wellness measures to assess the spiritual component of health, as well as having few positive measures in existing Indigenous wellness measures, highlighted the need to shift to strengths-based approaches [31,36,39,40]. For example, the article by Yates et al. highlighted that wellness indicators require a shift in focus “from reliance on narrow single-issue indicators, such as a country’s GDP, to broader collections of indicators that might account for health and wellbeing both now and into the future” [31]. In applying non-Indigenous indicators, wellness measurement neglected specificity in favor of superficial standards. Yates et al. further elaborated that the apparent quantitative nature of many [wellness] indicators can make the underlying concepts appear to be scientifically derived when they are actually culturally embedded preferences [31]. The cautions of deficit-focused wellness measures are relevant not only globally, but for multicultural nation states as well [29,32].

The articles highlighted that the prevailing narrative in governmental and academic reports consistently emphasize concerning issues such as high suicide rates, solvent abuse, and crime within Indigenous communities [29,34,43]. The majority of Indigenous participants in these projects found that deficit-focused reports often overlooked or failed to analyze the inherent joy and integrity that characterize their communities [38,41]. Instead, there is a disproportionate focus on the challenges and illnesses affecting Indigenous communities, neglecting to recognize their resilience, cultural strengths, and positive attributes [29,32,35]. This imbalance perpetuates a skewed perception that undermines a comprehensive understanding and portrayal of Indigenous life and culture. In order to shift to strengths-based wellness measures and indicators, a multidimensional and holistic understanding of wellness is critical to improving the overall wellbeing of Indigenous Peoples [29,30]. For example, McClintock et al. stated that an Indigenous worldview must “include individual wellbeing (including physical, mental, and emotional dimensions), relationships between people (including family, kinship or Tribal, and community dimensions), culture (including language, cultural knowledge, and cultural practice dimensions), and spirit (including spirit and Land dimensions)” [29].

Indigenous community members often expressed that they are tired of being viewed solely through a deficit-focused lens that neglects the interconnected relationships of wellness domains [29,32,33,35]. Many of the articles included large-scale wellness indicators set by non-Indigenous governments or organizations and neglected the interconnectedness of Indigenous wellness, thereby reducing and stripping a multitude of the inherent strengths embedded within Indigenous communities and cultures [42]. Indigenous community members stressed the importance of defining health and wellness for themselves, and the need to determine its importance to their quality of life [30,38,39]. A number of authors recognized that research conducted within a community partnership context identified a central tenet—the crucial role of autonomy and self-determination [31,37,41]. Autonomy and self-determination foster strengths-based approaches within Indigenous wellness research partnerships. Furthermore, upholding Indigenous self-determination and autonomy within wellness research partnerships helps to address the historical and ongoing impacts of colonization. Promoting and securing genuine autonomy and agency not only stands as a primary objective, but also forms a vital framework for addressing the complex challenges confronting Indigenous communities. With this, a strengths-based perspective emphasizes resilience, cultural strengths, and positive outcomes, countering narratives that disproportionately highlight the deficits and challenges within Indigenous contexts.

## 4. Discussion

Our scoping review identified five main categories in relation to measures of wellness that have been developed through Indigenous partnerships. These categories included the following: building relationships that uphold Indigenous worldviews (e.g., meaningful engagement and partnerships); a call for co-development protocols that weave multiple worldviews (e.g., using Indigenous methodologies and methods); needing awareness of the limitations in measuring Indigenous wellness (e.g., diversity of Indigenous Peoples, being inclusive of multiple dimensions of wellness); that the community-specific context is important (e.g., Indigenous ways of knowing and power dynamics); and a call for including strengths-based indicators (e.g., Indigenous Peoples currently being over-studied and over-exploited and Western society’s focus on deficit-based measures).

The review identified several key findings and implications for research and practice in relation to Indigenous wellness measures that have been developed through Indigenous partnerships. Out of the eighteen articles included in the review, only four underwent formal validation processes [30,39,40,42]. Despite this, many of the articles identified or proposed indicators for Indigenous wellness measures, highlighting a growing interest and effort in this critical area of study. The question of whether or not Western validation methods for measures are even appropriate is an outstanding question that needs to be considered within scholarship spaces. One notable approach discussed was the “shared space” method [38], exemplified by a study that integrated community, government, and scientific partners throughout the research process [30]. This collaborative approach ensured inclusivity and comprehensibility across all involved groups and combined grassroots involvement with a top-down direction to integrate scientific rigor, community engagement, and policy relevance effectively [30]. It also served as a capacity-building initiative, enhancing local capabilities and expertise within Indigenous communities [30].

Another observation from the included articles is that contemporary Western evidence-based practices overly prioritize Western methodologies and scientific rigor in research, often at the expense of incorporating more inclusive and self-determining Indigenous research methodologies and ethical frameworks [31,36,39]. Yet, these Indigenous frameworks are stated to be essential for ensuring that research processes are culturally safe, respectful, responsible, and beneficial to Indigenous communities. There is apprehension about the ethical implications of evidence-based practice [42], which sometimes fails to adequately consider the socially constructed nature of evidence production and review [29,36,43]. In essence, while evidence-based approaches were promoted as a means to improve outcomes, there was a growing recognition of their limitations in accommodating diverse cultural perspectives and ensuring equitable research practices for Indigenous communities [29,39,43]. Therefore, there is a need for a more nuanced approach that respects and integrates Indigenous Knowledge Systems and ethical standards into a broader framework of evidence-based practice in Indigenous measures of wellness research.

The use of Indigenous research methods such as Yarning, which fostered conversation and knowledge exchange grounded in Indigenous ways of doing, was notably scarce outside of Australia-based articles [37,39,43,45,46]. This lack of the use of Indigenous research methods across the included articles points to the need for the broader adoption of culturally appropriate assessment tools and participatory action research methods that prioritize Indigenous voices throughout all stages of research—from design to implementation and evaluation. The context within Australia appeared to be conducive to such inclusive methodologies for working with Indigenous measures of wellness, as evidenced by the included publications that highlighted culturally responsive approaches that centered Indigenous perspectives [37,39,43,45,46]. In contrast, the included articles outside of Australia often relied on non-Indigenous methodologies that may not have adequately captured the holistic nature of Indigenous wellbeing. There is clearly a need for more two-way learning approaches that foster more equitable partnerships, which could pave the way for the respective inclusion of Indigenous ways of knowing.

An additional observation from the included articles on Indigenous wellness measures was the limited inclusion of intergenerational engagement. Despite the often explicit recognition of the necessity for intergenerational engagement across all included articles, only a minimal number explicitly incorporated youth and Elder perspectives [39,41,42]. For example, while three articles addressed or involved Elders [39,40,42] and two involved young people [41,44], the predominant focus of the articles remained with adults. Intergenerational engagement is crucial for several reasons, including the need to ensure a holistic understanding of Indigenous wellbeing by encompassing perspectives across different age groups within the community [29,34,36,43]. Elders bring traditional knowledge and cultural continuity, while youth perspectives provide insights into contemporary challenges and aspirations [39,40]. By integrating these diverse viewpoints, research endeavors can better capture the multifaceted nature of Indigenous wellness and foster sustainable strategies for improvement. The scarcity of research around meaningful Indigenous wellness indicators further underscores the urgency of enhancing intergenerational Indigenous involvement in the development and refinement of wellness indicators overall. Including young people in research processes not only empowers younger generations, but also enriches studies with dynamic perspectives that are essential for addressing current and future health needs.

While the reviewed articles generally exemplified or included aspects of cultural relevance, community leadership, relationship building, and equitable engagement in Indigenous wellness partnerships, research outside of the reviewed articles still often falls short of effectively including Indigenous community-based approaches [32,33,37,38]. This review identified key findings for bridging this gap and advancing culturally responsive methodologies in Indigenous wellness research (e.g., utilizing a culturally rooted approach and building ongoing relationships) [29,32,33,40]. The included articles may, therefore, provide some reflection for other research projects engaging with measures of Indigenous wellness.

Overall, the articles advocated strongly for cultural relevance and community leadership in Indigenous wellness research [31,39,40]. The articles also demonstrated the critical need to mitigate the current dominance of external expertise by prioritizing Indigenous voices throughout all stages of research. Building authentic relationships was identified as a pivotal strategy to foster trust, mutual respect, and genuine collaboration [29,31,35]. To address Indigenous wellness research challenges, future research around Indigenous measures of wellness must prioritize actionable steps to operationalize cultural safety and responsiveness. This includes adopting participatory action research frameworks that are inclusive of Indigenous communities in decision-making processes and uplifting Indigenous knowledge systems [30,38,39,40,43,44]. Such participatory approaches not only enhance the robustness of research outcomes, but also contribute to more sustainable health interventions that align with Indigenous values and aspirations.

While progress is occurring in the development and recognition of Indigenous wellness measures, there remains significant work ahead to ensure that research methodologies honor and align with Indigenous ways of being and doing. Continued efforts to integrate Indigenous perspectives and methodologies are crucial for advancing meaningful engagement and improving wellness outcomes for Indigenous Peoples worldwide. The discrepancies between the articles included in our review and broader public health research practices highlight the urgent need for capacity-building initiatives and policy frameworks that support culturally responsive methodologies. Non-Indigenous organizations and governments must commit to transformative practices that recognize and uphold Indigenous rights, perspectives, and self-determination.

### Limitations and Strengths

Given the varied use of terms used to encompass the meaning of wellness (e.g., wellbeing, health, quality of life, etc.), as well as the multitude of potential terms for Indigenous Peoples around the world (e.g., specific Indigenous Nations or community names, etc.), there is a possibility that our search strategy missed relevant perspectives on measures of wellness that have been developed through Indigenous partnerships. Additionally, as we were only examining a certain sub-set of what may constitute a measure of wellness in various Indigenous communities, broader research should be engaged to better appreciate the body of work on this topic area outside of our review criteria. For example, many Indigenous communities pass on knowledge orally and not through written texts or documents, which can structurally limit Indigenous project work’s ability to share findings. Nonetheless, the categories framed within this review will likely still be relevant when it comes to appreciating the care needed for the development of Indigenous measures of wellness through Indigenous partnerships. We only included articles in this review that were published in English, which could have also limited the articles relevant to the review question. The inclusion of articles relevant to Indigenous populations globally may also limit the applicability of the findings to individual Indigenous communities. Despite these limitations, as this is the first review examining measures of wellness through Indigenous partnerships, we feel that we have captured the key categories that may support future work in this area. The articles included in the review identified meaningful approaches for engaging Indigenous populations in conversations on measures of wellness across various geographic domains. Although the results may not be applicable to all regions, they contain key considerations for reflecting on measures of Indigenous wellness more broadly. Scoping reviews are limited in nature, as they aim to provide a high-level overview of a specified research topic, compared to systematic reviews, which aim to acquire all evidence of a given subject [47]. Within this scoping review, therefore, we did not go into fine detail on aspects of the categories that might be common in other types of review formats. The intent of the scoping review was to identify the overarching categories reflected in the current literature on this topic. The review is intended to be inclusive of some of the key features important in advancing Indigenous measures of wellness work in the future.

## 5. Conclusions

We reviewed the literature to identify common approaches for engaging Indigenous populations in conversations on measures of wellness. The review highlighted persistent challenges in current wellness measures that often overlook or misrepresent Indigenous experiences. These limitations stem from the historical neglect of Indigenous knowledge systems and the imposition of Western-centric frameworks that fail to capture the holistic nature of Indigenous wellness. By emphasizing community-specific contexts and advocating for strengths-based indicators, researchers can foster a more nuanced understanding of Indigenous wellness that celebrates resilience, cultural strengths, and positive attributes. Building relationships grounded in mutual respect and co-developing engagement protocols are crucial steps towards dismantling power imbalances and promoting inclusive research practices that uplift Indigenous communities. The review also highlighted that there is a critical need to co-develop meaningful engagement protocols through respectful Indigenous partnerships, as well as community-relevant and meaningful wellness indicators. Further collaboration and respectful partnerships between Indigenous and non-Indigenous people and organizations may lead to the co-development and integration of more community attuned Indigenous methodological and culturally responsive approaches for measuring wellness. Wellness for Indigenous Peoples is often deeply intertwined with their identities, cultures, and ways of life, all of which profoundly impact their physical and spiritual wellbeing. Developing collaborative efforts for measuring wellness within Indigenous communities may garner greater community support, help to influence institutional systems, and catalyze changes in policies and practices. Our scoping review provides further evidence that there is an urgent need to center Indigenous worldviews and knowledge systems in all efforts attempting to measure wellness within Indigenous communities.

### Future Directions

To advance Indigenous wellness research effectively, several strategic directions must be pursued. Firstly, there is a pressing need to prioritize cultural relevance and autonomy in research methodologies. This involves platforming Indigenous ways of knowing and ethical frameworks throughout all stages of research, ensuring that studies are not only respectful, but also beneficial and meaningful to Indigenous communities. By centering Indigenous voices and perspectives, researchers may contribute to more accurate and culturally sensitive wellness measures. Secondly, enhancing community partnership and engagement is essential. Future research should prioritize inclusive and participatory approaches that involves diverse community rights-holders, including Elders and young people. These partnerships are crucial for ensuring that research outcomes reflect the multifaceted dimensions of Indigenous wellness and lead to sustainable improvements in community health and wellbeing. By embracing these strategic directions—cultural relevance, community engagement, and strengths-based approaches—researchers can pave the way for transformative advancements in Indigenous wellness measures. This Indigenous-informed approach not only respects Indigenous autonomy and self-determination, but also facilitates meaningful collaboration that bridges cultural divides and promotes holistic health outcomes for Indigenous Peoples worldwide.

## Figures and Tables

**Table 1:** Concept levels of Indigenous wellness *.

Wellness Circle	Description
Center Circle	Human Being
Second Circle	Mental, Emotional, Cultural, and Economic
Third Circle	Respect, Wisdom, Responsibility, and Relationships
Fourth Circle	Land, Community, Family, and Nations
Fifth Circle	Social, Environmental, Cultural, and Economic

* Adapted from the First Nation Health Authority (FNHA) framework of First Nation Perspectives of Health and Wellness [26].

**Table 2:** Characteristics of the included articles of the review.

Reference	Year	Country	Study Design *	Setting	Key Findings Partnership Context	Key Findings Measures of Wellness
*Jeffery* et al. [33]	2006	CA	Qual	Urban	This study examines community engagement in the creation of health and capacity indicators. It builds on earlier collaborations from 2002 and ongoing partnerships with local health authorities in northern Saskatchewan. A key focus was a comprehensive review of the current population’s health indicators and frameworks, revealing gaps in culturally relevant measures. The initiative also sought to develop practical methods for local health organizations to implement and track these indicators, ensuring that they meet community needs effectively.	Wellness is strengthened through sustainable land use practices and preserving natural resources for future generations. Healthy ecosystems, supporting diverse life forms, and avoiding extinction risks promote wellness. Community wellness thrives in environments with strong social support networks, fostering collective wellbeing. The equitable distribution of wealth through economic activities that meet community needs enhances wellness, perceived as fair and just by the majority.
*McCubbin* et al. [40]	2013	US	MM	Urban/Rural	This research focused on developing a relational wellbeing measure for Indigenous Native Hawaiians through collaboration with local communities. Partnerships with Indigenous groups were vital for ensuring cultural relevance and inclusivity in the measure’s development. The process aimed to capture diverse experiences while avoiding reductive interpretations of Indigenous culture. The investigation underscores the importance of respecting individuals’ cultures and values as a foundation for assessing relational wellbeing, with a commitment to identifying factors that promote physical, psychological, and interpersonal health.	Indigenous Relational Wellbeing is characterized by satisfaction and happiness derived from confidence, competence in overcoming adversity, respect, and connection with nature and ancestors through cultural practices, financial management, family commitment, healthcare access, and community involvement. This research critiques the Western–European wellbeing paradigm, arguing that it is inadequate for evaluating the wellbeing of individuals and families in Indigenous cultures. It highlights the importance of values such as respect for ancestors, cultural traditions, harmony with nature, resource management, cultural and language preservation, and collectivism as essential components of wellbeing.
*Young* et al. [42]	2013	CA	MM	Rural	This study showcased a collaborative approach to data collection that emphasizes community involvement and cultural relevance. By utilizing photovoice informed by an Aboriginal framework, participants engaged actively in the research process through a method that resonates with their experiences. The synthesis of concepts into research items through expert consensus, followed by community review, demonstrated a commitment to inclusivity and collective input, reinforcing the significance of partnership in producing meaningful research outcomes.	A total of 38 participants identified 206 key wellness concepts, primarily derived from children and young people. The remaining wellness concepts came from questionnaires and the community. While aligned with the four quadrants of the Medicine Wheel—emotional, spiritual, physical, and mental—the participants noted that some wellness concepts fit into multiple domains. A total of 17 concepts were categorized into two or three domains. Additionally, 25 concepts were placed in an “overall” domain, reflecting all four areas, sourced from various contributions.
*Cairney* et al. [34]	2014	AU	MM	Rural	The Interplay project utilized a grounded theory approach to develop a comprehensive wellbeing framework, analyzing data through constant comparison methods from diverse participants. Community initiation fostered extensive engagement via workshops, visits, and active involvement of Aboriginal researchers in refining methodologies. The team’s diverse expertise, blending disciplines like neuroscience, psychology, biostatistics, Aboriginal wellbeing, education, ecology, and evaluation, included roughly half Aboriginal members.	Cultural Continuity and Identity are critical for fostering positive identity and overall wellbeing, though balancing cultural obligations with mainstream systems presents challenges that impact mental and emotional health. Strong community and family ties play a vital role in wellbeing, promoting resilience and addressing challenges related to cultural preservation and identity. Despite obstacles such as limited cultural engagement opportunities and historical policies, resilience is demonstrated through ongoing efforts to maintain and adapt cultural practices to contemporary contexts.
*Yap* et al. [35]	2016	AU	Qual	Urban	This partnership exemplifies community involvement from project initiation to dissemination. It originated from a shared interest in measuring wellbeing through Yawuru worldviews, connecting a PhD proposal by a non-Indigenous student with Yawuru goals for culturally relevant measures. Yawuru participation was critical at every stage, including the formation of a steering committee of Elders to ensure that local values guided the research. This collaboration enabled the development of culturally grounded indicators through semi-structured interviews and capacity-building for Yawuru research assistants in the quantitative phase. The qualitative component gathered Yawuru perspectives on a good life through interviews and focus groups, emphasizing the significance of a participatory approach for community-driven research outcomes.	The interviews identified central themes of wellbeing among the Yawuru, focused on “mabu liyan” (good life) and the holistic feeling of “liyan”, which arises from sensory experiences, relationships, and cultural practices. Family and community are crucial in fostering “liyan” through support and maintaining connections to culture and Land. Access to resources and autonomy are important for participating in cultural activities and securing livelihoods. However, ongoing socio-historical challenges, including institutional restrictions and the effects of past policies like the Stolen Generation, continue to impact Yawuru wellbeing, underscoring the struggle for cultural continuity and autonomy.
*Cairney* et al. [30]	2017	AU	MM	Rural	The Interplay project employed a collaborative approach, engaging both Aboriginal and non-Aboriginal researchers through workshops focused on methods, wellbeing, and relevant tool development. The team includes approximately half Aboriginal members, bringing diverse expertise. Community engagement involved over 60 Aboriginal researchers and interactions with more than 100 remote communities, ensuring that the framework aligned with local needs. A national advisory group, emphasizing end-user and Aboriginal representation, contributed to refining the research approach, ensuring responsiveness to community needs and potential impacts.	Cultural connections to Land, family, and traditional knowledge are central to Aboriginal wellbeing, as illustrated by the “Tree of Wellbeing” model, which links various life components. Cultural obligations often take precedence over mainstream activities, leading to conflicts that can hinder participation in education and work. Language plays a vital role in identity and connection, yet many face challenges in its use within mainstream contexts. Empowerment is essential for Aboriginal communities to influence decision making, with calls for two-way learning and greater local governance. Finally, quantifying cultural wellbeing remains challenging, highlighting the need for tailored measures that reflect Aboriginal perspectives.
*Peters* et al. [39]	2019	US	Quant	School, Rural/Tribal	The Wicozani Instrument was developed collaboratively by Dakota Wicohan research partners and a diverse research team, integrating Indigenous knowledge to ensure community relevance. This partnership emphasizes the importance of Indigenous epistemologies, with Elders and community members actively shaping the research process. By utilizing the instrument, the study empowered members to define their own health and wellbeing. The validation of the Wicozani Instrument affirmed its reliability as a culturally relevant tool for measuring health from an Indigenous perspective.	The Wicozani Self-Knowledge subscale focuses specifically on cultural awareness and understanding related to mental, physical, and spiritual health within the Dakota context. In contrast, general self-knowledge refers to broader self-awareness that may not incorporate cultural dimensions. In the findings, participants rate the importance of Wicozani to their quality of life significantly higher than their self-knowledge scores, indicating that cultural understanding and connection (Wicozani) play a critical role in perceived wellbeing, suggesting that cultural identity and practices enhance overall wellness beyond just self-awareness.
*Leech* et al. [32]	2020	CA	Qual	Urban/Rural	The key findings in the partnership context highlight the initiative’s successful collaboration with various Aboriginal communities across Canada, including the Little Red River Cree Nation, Mohawk Community of Akwesasne, and others. This project emphasized the importance of ongoing engagement, showcasing a commitment to innovation and adaptability based on community feedback and evolving needs. This approach fostered trust and responsiveness, ensuring that the initiative remained relevant and effective in addressing the unique challenges faced by each community.	The key findings on measures of wellness indicate that values and beliefs, conveyed through stories, legends, and rituals, embody community ideals and behaviors, with interpretations varying across different communities. Responsibility is emphasized as fulfilling obligations to both the community and the environment. Spirituality plays a crucial role, fostering connections to the ‘Good Mind’ and the universe, and maintaining a balance among these spiritual elements is essential for enhancing overall community health.
*McClintock* et al. [29]	2021	NZ, AU, CA, US	Qual	Urban/Rural	The article highlights the collaboration of senior Indigenous researchers from Aotearoa, Australia, Canada, and the US within the International Group on Indigenous Health Measurement (IGIHM). This initiative aimed to create a global measure of Indigenous wellbeing informed by Indigenous Ways of Knowing and findings from national health surveys. The group addressed Indigenous data gaps and promoted a shared understanding of wellbeing across diverse Indigenous communities. Regular teleconferences supported knowledge exchange, enhancing health information collection for Indigenous populations.	The article identified the following four key measures of wellness: Physical, Kinship, Cultural, and Spiritual dimensions. Each dimension represents a vital aspect of wellbeing, encompassing specific measurable categories that emphasize the interconnectedness of these elements. This holistic framework illustrates how these dimensions collectively contribute to Indigenous perspectives on health and wellness, reinforcing the importance of relationships and cultural practices in promoting overall community wellbeing.
*Smith* et al. [38]	2021	AU	MM	Urban	Utilizing Participatory Action Research, this study involved interviews and yarning groups with participants over 45 years old in Perth and Melbourne to identify factors contributing to “a good life”. The resulting draft QoL tool, named the Good Spirit, Good Life tool, encapsulates twelve vital factors and will undergo further testing for validity. This culturally grounded approach emphasizes Elders’ involvement and can be adapted for other Indigenous populations globally, showcasing the effectiveness of Indigenous research methodologies in developing relevant wellbeing measures.	Participants preferred “a good life” over quality of life (QoL), highlighting that a good spirit is fundamental to this concept. Key protective factors for achieving a good life included family and friends, health, culture, the role of Elders, respect, connection to Country, spirituality, access to services and support, community involvement, future aspirations, safety and security, and meeting basic needs.
*Wright* et al. [41]	2021	AU	Qual	Urban	Through a comprehensive participatory action research initiative, Elders, service leaders, and both Aboriginal and non-Aboriginal researchers worked collaboratively to design evaluation tools aimed at assessing an engagement framework. This effort emphasized Aboriginal perspectives, as participants engaged in co-design workshops to ensure cultural relevance. The outcome was a tripartite survey developed to capture service experiences related to cultural safety, incorporating insights from Aboriginal clients, their caregivers, and the service staff they interact with.	The study highlights the essential role of family and community in understanding and integrating collective wellbeing and recovery experiences within Aboriginal communities. It emphasizes that relationships are central to individuals’ sense of belonging and wellness, supporting resilience amid social and emotional challenges. Furthermore, the research advocates for prioritizing meaningful measures over extensive data collection, stressing the importance of qualitative aspects that, while difficult to quantify, are vital to Aboriginal cultural perspectives.
*Bourke* et al. [37]	2022	AU	Qual	Urban/Rural	This study utilizes 28 focus groups to effectively capture the diverse perspectives of Aboriginal and Torres Strait Islander cultures. An iterative process refined the questionnaire based on insights from these groups, enhancing cultural relevance. Facilitated by an Aboriginal Mayi Kuwayu team member, sessions follow cultural protocols, including gender-specific discussions and the introduction of facilitators’ cultural backgrounds. Participants were informed about the study’s purpose and confidentiality measures, fostering open and respectful dialogue throughout the process.	The study emphasizes that, for Aboriginal and Torres Strait Islander Peoples, culture is essential to health and wellbeing, yet its complexity is often inadequately addressed in existing research. Through extensive community consultations, key cultural wellness domains were identified, including Connection to Country, Beliefs and Knowledge, Language, Family, Kinship and Community, Cultural Expression and Continuity, and Self-determination and Leadership. These findings underscore the need for measures that reflect the diversity and significance of Indigenous cultural perspectives, highlighting that understanding wellbeing requires meaningful cultural representation in health assessments.
*Macniven* et al. [36]	2022	AU	Qual	Urban	The study addresses the importance of physical activity for the health and wellbeing of Aboriginal and Torres Strait Islander Peoples, highlighting the need for the effective evaluation of related programs. A collaborative approach was taken to select measures for assessing the health impacts of Indigenous community running groups. Nine female participants from a regional New South Wales town shared their insights through the Indigenous research method of Yarning. This co-selection process not only enhanced the evaluation of physical activity programs, but also ensured that measures resonate with the community’s lived experiences, offering a model for broader application in future program evaluations.	Participants highlighted their holistic reasons for engaging in running activities, citing physical, mental, and social benefits. They emphasizede the importance of group participation for mutual social support. The identified measures related to physical activity, lifestyle, physical health, and social and emotional wellbeing receive strong endorsement. Furthermore, participants note the relevance of social networks and sports injuries in their overall experiences.
*Yates* et al. [31]	2022	NZ	Qual	NA	Indigenous scholars, along with community partners, are actively engaged in creating tools that incorporate Indigenous–Māori cultural perspectives, ensuring that these frameworks are culturally relevant and context-specific. There is a critical focus on integrating both human and more-than-human wellbeing into governance practices. The findings also caution against the risks of universalizing wellbeing indices, advocating for localized approaches that reflect the unique socio-ecological contexts of communities. Ultimately, these collaborative efforts aim to produce transformative metrics that effectively address current socio-ecological crises.	This emphasized the urgent need for responsive wellbeing-led governance frameworks that encompass both human and more-than-human wellbeing, particularly in the face of ecological emergencies. While existing frameworks have begun to include more-than-human wellbeing indices, there is caution against the risks of universalizing these approaches. The paper advocates for a pluriversal and prefigurative project where Indigenous scholars collaborate with partners to co-create culturally specific and holistic wellbeing tools. By integrating Indigenous–Māori perspectives, the initiative aims to develop metrics and tools that are both transformative and situated, contributing to more effective governance during socio-ecological crises.
*Gilchrist* et al. [43]	2023	AU	Quant	Urban	This study was guided by an Elders Governance Group and developed in partnership with older Aboriginal Australians, ensuring that the GSGL tool reflected Aboriginal perspectives on wellbeing. Aboriginal researchers led data collection and analysis, enhancing cultural safety and trust. The tool was designed to capture the holistic quality of life dimensions, including connection to Country, spirituality, and community. Recommendations emphasized the need for service providers to support connection to Country and build trusting relationships with Aboriginal communities. Ongoing validation is planned for remote populations and Torres Strait Islander Peoples, highlighting the importance of culturally relevant measures in future research.	The study identified key wellness measures through the Good Spirit, Good Life (GSGL) tool, which emphasizes holistic wellbeing for older Aboriginal Australians. Factor analysis revealed the following two key components: foundation (family, community, culture, spirituality, Elder role, safety, and Country) and external (health, support, services, future planning, and basic needs). Items like connection to Country, Elder roles, and respect were most strongly correlated with overall wellbeing. The tool also highlighted unmet needs, particularly around connection to Country. Additionally, mental health (anxiety and depression) significantly impacted wellness, with lower GSGL scores recorded among those with these conditions. The tool’s validity across different settings (urban and regional) suggests that it captured essential wellness aspects specific to the Aboriginal worldview.
*Stelkia* et al. [44]	2023	CA	Qual	N/A	The article emphasizes the importance of meaningful partnerships in developing Indigenous health indicators. It advocates for community-driven, culturally relevant, and holistic approaches that reflect Indigenous worldviews. Key to this process is ensuring full Indigenous leadership and participation at all stages, from indicator development to interpretation and reporting. Establishing respectful, ongoing relationships with communities ensures that the indicators are not only relevant, but also grounded in local knowledge. Effective partnerships also requires that Indigenous Peoples lead their health narratives, ensuring that reporting practices honor Indigenous knowledge systems and methodologies.	Effective Indigenous health indicators must encompass a holistic view of wellness, integrating physical, mental, emotional, and spiritual dimensions. Western indicators often fail to capture these aspects, focusing primarily on physical health. To measure wellness more accurately, indicators should be grounded in Indigenous worldviews, incorporating elements such as connection to land, culture, and community. These measures must adopt a strengths-based approach, acknowledging both resilience and health challenges, and should be community-defined and validated to reflect Indigenous values and priorities.
*Howard* et al. [45]	2024	AU	MM	Urban/Rural	The development of the What Matters 2 Adults (WM2A) measure was driven by strong partnerships with Aboriginal and Torres Strait Islander communities, involving key governance bodies such as the Indigenous Project Advisory Group (IPAG) and Indigenous Researchers Group (IRG). These groups provided cultural oversight, ensuring the measure reflected Indigenous worldviews and values. The process integrated collaborative, strengths-based approaches through Yarning Circles and ongoing feedback, ensuring the measure’s cultural relevance and accuracy. This partnership model combined Indigenist methodologies with traditional psychometric methods to develop a measure that is both scientifically rigorous and culturally appropriate.	The WM2A measure represents a holistic understanding of wellness, rooted in Aboriginal and Torres Strait Islander conceptions of wellbeing. It moves beyond traditional, Western-defined quality of life measures by capturing dimensions such as belonging, connection, dignity, respect, and community. Through Yarning Circles and conceptual framework development, key domains like family, culture, and access to services were identified as central to wellbeing. The measure used item and factor analysis to ensure that it effectively captured these interconnected wellness aspects. The final 10-dimensional structure reflects both individual and collective wellbeing, acknowledging the interdependence of health, culture, and community.
*Meldrum* et al. [46]	2024	AU	Quant	Urban/Rural	This Delphi study emphasized the importance of collaboration with both Indigenous and non-Indigenous experts in mental health and social and emotional wellbeing. It involved a diverse group of stakeholders, including researchers, clinicians, and community members, ensuring that cultural perspectives from the Torres Strait were integral to the development of the new Social and Emotional Well Being (SEWB) screening tool. Feedback from the Yarning circles was also central to the tool’s design, highlighting the value of community-driven insights and expertise in creating culturally relevant health tools.	The Delphi study confirms that existing screening tools, such as the PHQ-9 and KICA-Dep, were not fully suitable for assessing the social and emotional wellbeing (SEWB) in First Nations Peoples of the Torres Strait and NPA. The preference was for a tool that incorporated culturally relevant language and concepts, emphasizing the importance of a Yarning approach for screening. Participants suggested adapting existing tools by integrating local terms and visual scales, such as faces or color gradients, to better reflect emotional states. There was also support for a combined tool that could use both open-ended Yarning and standardized, quantitative measures to ensure a holistic assessment of wellbeing.

* Study Design abbreviations: Qual (Qualitative), Quant (Quantitative), and MM (Mixed Methods).

**Table 3:** Overview of the main categories identified for engaging Indigenous populations in conversations on measures of wellness through Indigenous partnerships.

Characterized Categories
1. Building relationships that uphold Indigenous worldviews necessitates community-driven processes for measuring wellness
2. There is a need to move towards co-development engagement protocols that weave multiple worldviews
3. Awareness is needed about some of the limitations in measuring wellness within Indigenous community settings
4. Community-specific context matters when developing Indigenous indicators and measures of wellness
5. There is a need to shift to strengths-based Indigenous wellness measures and indicators

## Data Availability

The original contributions presented in this study are included in the article/Supplementary Material. Further inquiries can be directed to the corresponding authors.

## Supplementary Materials

The following supporting information can be downloaded at: https://www.mdpi.com/article/10.3390/ijerph22010043/s1, File S1: Search Strategy; File S2: Summary Results of Publications Included in the Scoping Review.
