# Supplementary material for: Measuring Wellness Through Indigenous Partnerships: A Scoping Review
Source: Int J Environ Res Public Health. 2024 Dec 31;22(1):43. doi: 10.3390/ijerph22010043 (PMC11764526; doi:10.3390/ijerph22010043)
Supplement: Supplementary file 1 [file ijerph-22-00043-s001.zip › S1 file. Search Strategy.pdf]

S1 file. Search Strategy

Table S1. Example PubMed Search Strategy

**PubMed Search Strategy**
(((evaluation OR development) n3 (instrument OR questionnaire OR framework OR protocol)) OR "Validation Study"[pt] OR "Psychometrics"[MAJR] OR "Self Report"[MAJR] OR "Program Evaluation"[MAJR] OR "Health Services, Indigenous"[MAJR]) (indigenous[Title] OR aborigin*[Title] OR "Native American"[Title] OR tribal[Title] OR "American Indian"[Title] OR "remote community"[Title] OR "Alaska Native"[Title] OR "Pacific Islander"[Title] OR Amerind*[Title] OR Inuit[Title] OR Innuit*[Title] OR Inuk[Title] OR Inuktitut-speaker*[Title] OR Inupiat*[Title] OR "First Nation"[title] OR "First Nations"[Title] OR "First Peoples"[Title] OR Metis[Title] OR tribe[Title] OR tribes[Title] OR tribal[Title] OR "American Native Ancestry Group"[Mesh] OR "Native Hawaiian or Other Pacific Islander"[Mesh] OR "Indigenous Peoples"[Mesh]) (wellbeing[Title] OR well-being[Title] OR wellness[Title] OR"Quality of Life"[MAJR]entry 2
